# Examining the longitudinal nature of depressive symptoms in the Avon Longitudinal Study of Parents and Children (ALSPAC)

**DOI:** 10.12688/wellcomeopenres.15395.2

**Published:** 2019-10-04

**Authors:** Alex S. F. Kwong

**Affiliations:** 1 School of Geographical Sciences, University of Bristol, Bristol, UK; 2 MRC Integrative Epidemiology Unit, University of Bristol, Bristol, UK

**Keywords:** longitudinal, depression, depressive symptoms, ALSPAC

## Abstract

Depression during adolescence is associated with a number of negative outcomes in later life. Research has examined the longitudinal nature of adolescent depression in order to identify patterns of depressive mood, the early antecedents and later consequences. However, rich longitudinal data is needed to better address these questions. The Avon Longitudinal Study of Parents and Children (ALSPAC) is an intergenerational birth cohort with nine repeated assessments of depressive symptoms throughout late childhood, adolescence and young adulthood. Depressive symptoms are measured using the Short Mood and Feelings Questionnaire (SMFQ). Many studies have used ALSPAC to examine the longitudinal nature of depressive symptoms in combination with the wealth of early life exposure and later outcome data. This data note provides a summary of the SMFQ data, where the data are stored in ALSPAC, the characteristics and distribution of the SMFQ, and highlights some considerations for researchers wanting to use the SMFQ data in ALSPAC.

## Introduction

Depression during adolescence is associated with a number of negative outcomes in later life such as poorer mental health ^1^, impaired educational attainment ^2^ and reduced social functioning ^3^. Understandably, research has examined the aetiology of depression during and around adolescence in order to identify preventions and interventions that could reduce these impairments.

Adolescence marks a period where depression as a disorder first commonly onsets ^4^, but this period is also characterised by dynamic changes in depressive mood ^5^. Consequently, depression during and surrounding adolescence can fluctuate rapidly across short periods of time ^6^, and it can be difficult to quantify the true nature of adolescent depression without longitudinal research. Several recent studies have suggested that examining depression within individuals over time may be helpful method for 1) uncovering the nature of adolescent depression and how it changes over time, 2) identifying risk factors associated with greater adolescent depression, and 3) examining how greater depression during and across adolescence is associated with later outcomes ^7^.

The Avon Longitudinal Study of Parents and Children (ALSPAC) is a unique intergenerational cohort study with a wealth of biological, genetic and phenotypic data from parents and children. However, one of the most important aspects of the ALSPAC study is through its repeated assessments of psychiatric traits ^8^. ALSPAC is one of the few cohorts that has repeated assessments of the Short Mood and Feelings Questionnaire (SMFQ) ^9^, reported by the child themselves throughout childhood, adolescence and young adulthood. The SMFQ is a 13-item questionnaire designed for examining the presence of depressive symptoms in epidemiological studies ^9– 11^, and has been shown to be a strong predictor of depression ^12^. The SMFQ summaries these 13 items to give a score ranging between 0–26, where greater scores represent higher depression. 

Many studies have used to examine the SMFQ in ALSPAC for exploring the nature of depression across adolescence ^6^, risk factors for greater depression ^13– 16^, and how depression during this period can be associated with later outcomes ^2, 17^. However, there are still many unanswered questions regarding the longitudinal nature of depression during and across adolescence, and ALSPAC will play a role in answering these questions. Therefore, the aim of this data note is to provide the reader with a comprehensive overview of the SMFQ in ALSPAC. Primarily, this data note focuses on the location of these data within ALSPAC, the sample sizes, validity and correlations between SMFQ assessments and the distribution of the SMFQ data.

## Methods

### ALSPAC data

The Avon Longitudinal Study of parents and Children (ALSPAC) is an intergenerational longitudinal cohort that recruited pregnant women residing in Avon, UK with expected dates of delivery 1 ^st^ April 1991 to 31 ^st^ December 1992 ^18, 19^. The initial cohort consisted of 14,062 children, but has been increased to 14,901 with further recruitment ^20^. The study website contains details of all the data that are available through a fully searchable data dictionary and variable search tool: http://www.bristol.ac.uk/alspac/researchers/our-data. Part of the depressive symptoms data were collected using REDCap.

### The SMFQ

The SMFQ is a 13-item questionnaire that measures the presence of depressive symptoms in the last two weeks ^9^. Table 1 shows the list of questions administered at each occasion in ALSPAC. For each question, the answer can be “not true” (scored 0), “sometimes” (scored 1) and “true” (scored 2). As each question is scored between 0–2, the resulting summary score of all the items can range between 0–26, with higher scores being more indicative of greater depression. As well as being used as a dimensional outcome, some studies have also shown that a cut off point for scores of 11 or more have good specificity for predicting depression ^12^. As such, this binary threshold has also been used in several studies ^10, 13^.

**Table 1.** List of questions in the Short Mood and Feelings Questionnaire.

Question number	List of questions used
1	I felt miserable or unhappy
2	I didn’t enjoy anything at all
3	I felt so tired I just sat around and did nothing
4	I was very restless
5	I felt I was no good anymore
6	I cried a lot
7	I found it hard to think properly or concentrate
8	I hated myself
9	I was a bad person
10	I felt lonely
11	I thought nobody really loved me
12	I thought I could never be as good as others
13	I did everything wrong

For each question, the responses are: not true (scored 0), sometimes (scored 1) and true (scored 2). The total scores are then added up to give a score ranging between 0 and 26 where higher scores indicate higher depressive symptoms.

### SMFQ within ALSPAC

The SMFQ has been measured on nine occasions between the ages of 10 and 24 in the ALSPAC cohort. At each of these occasions, the SMFQ has been self-completed by the child/young person. However, there are an additional four occasions where the SMFQ has been completed by a parent or guardian for the child/young person; these data are not the subject of this data note.

The SMFQ was administered in ALSPAC via postal/email questionnaire or at research clinics. Table 2 shows the how each questionnaire was collected. Across the nine occasions, the SMFQ has been collected via post/email on five occasions, and via a research clinic on the four other occasions. ALSPAC data is split between questionnaire files (post/email) and clinic files; Table 2 also highlights the name of the files where the SMFQ data is stored, along with the names of the SMFQ questions. Syntax for creating the scores is provided as *Extended data* ^21^.

**Table 2.** Source of Short Mood and Feelings Questionnaire (SMFQ) questions in ALSPAC and variable names.

Occasion	Source of SMFQ	Source file in ALSPAC	List of variable names in ALSPAC
1	Clinic	Focus at 10 (F10)	fddp110, fddp112, fddp113, fddp114, fddp115, fddp116, fddp118, fddp119, fddp121, fddp122, fddp123, fddp124, fddp125
2	Clinic	Teen Focus 1 (TF1)	ff6500, ff6502, ff6503, ff6504, ff6505, ff6506, ff6508, ff6509, ff6511, ff6512, ff6513, ff6514, ff6515
3	Clinic	Teen Focus 2 (TF2)	fg7210, fg7212, fg7213, fg7214, fg7215, fg7216, fg7218, fg7219, fg7221, fg7222, fg7223, fg7224, fg7225
4	Questionnaire	CCS	ccs4500, ccs4502, ccs4503, ccs4504, ccs4505, ccs4506, ccs4508, ccs4509, ccs4511, ccs4512, ccs4513, ccs4514, ccs4515
5	Clinic	CCXD (TF4) *	CCXD900, CCXD902, CCXD903, CCXD904, CCXD905, CCXD906, CCXD908, CCXD909, CCXD911, CCXD912, CCXD913, CCXD914, CCXD915
6	Questionnaire	CCT	cct2700, cct2701, cct2702, cct2703, cct2704, cct2705, cct2706, cct2707, cct2708, cct2709, cct2710, cct2711, cct2712
7	Questionnaire	YPA	YPA2000, YPA2010, YPA2020, YPA2030, YPA2040, YPA2050, YPA2060, YPA2070, YPA2080, YPA2090, YPA2100, YPA2110, YPA2120
8	Questionnaire	YPB	YPB5000, YPB5010, YPB5030, YPB5040, YPB5050, YPB5060, YPB5080, YPB5090, YPB5100, YPB5120, YPB5130, YPB5150, YPB5170
9	Questionnaire	YPC	YPC1650, YPC1651, YPC1653, YPC1654, YPC1655, YPC1656, YPC1658, YPC1659, YPC1660, YPC1662, YPC166,3 YPC1665, YPC1667

*Note, the SMFQ was assessed at the teen focus 4 clinic (TF4) but was released in a separate questionnaire file (CCXD).

The SMFQ in ALSPAC was not collected at regular age intervals. Table 3 shows the mean age of participants at each assessment. There is no obvious pattern for time between assessments but the longest period between assessments falls between the ages of 18.6 and 21.95 years. The shortest period between assessments falls between the ages of 17.84 and 18.65.

**Table 3.** Descriptive statistics and reliability of the Short Mood and Feelings Questionnaire (SMFQ).

Occasion	Mean Age	Sample Size	SMFQ Mean	SMFQ SD	SMFQ Median	SMFQ IQR	% Above SMFQ Threshold (≥11)	*α*
1	10.65	7,364	4.04	3.51	3	5	5.96%	0.797
2	12.81	6,716	3.97	3.86	3	4	7.10%	0.842
3	13.84	6,019	4.92	4.49	4	5	11.66%	0.865
4	16.68	4,997	5.91	5.64	4	6	18.05%	0.908
5	17.84	4,497	6.59	5.25	5	7	21.64%	0.897
6	18.65	3,335	6.83	5.93	5	8	21.86%	0.906
7	21.95	3,305	5.70	5.58	4	6	18.06%	0.915
8	22.88	3,856	6.21	5.55	5	7	18.80%	0.906
9	23.80	3,915	7.03	6.06	5	8	24.75%	0.913

SD: Standard deviations; α: coefficient alpha estimate of reliability for the SMFQ at each occasion. The SMFQ ranges between 0–26 and scores of, or exceeding 11 have been proposed as good indicators for a diagnosis of depression ^12^.

### Characteristics of the SMFQ in ALSPAC

The sample size of the SMFQ also tends to vary in ALSPAC, with a maximum sample of 7,364 at the first occasion (age 10.65), compared to the lowest sample of 3,305 at the seventh occasion (age 21.95). Note that sample size has increased in the latter waves of data collection. However, the overall trend of decreasing sample size means that researchers should be aware of this attrition and take steps towards addressing it such as multiple imputation or full information maximum likelihood.

**Figure 1.**
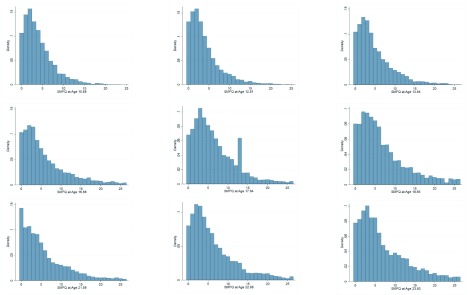
Histograms for the Short Mood and Feelings Questionnaire (SMFQ) at each of the nine occasions in ALSPAC.

**Figure 2.**
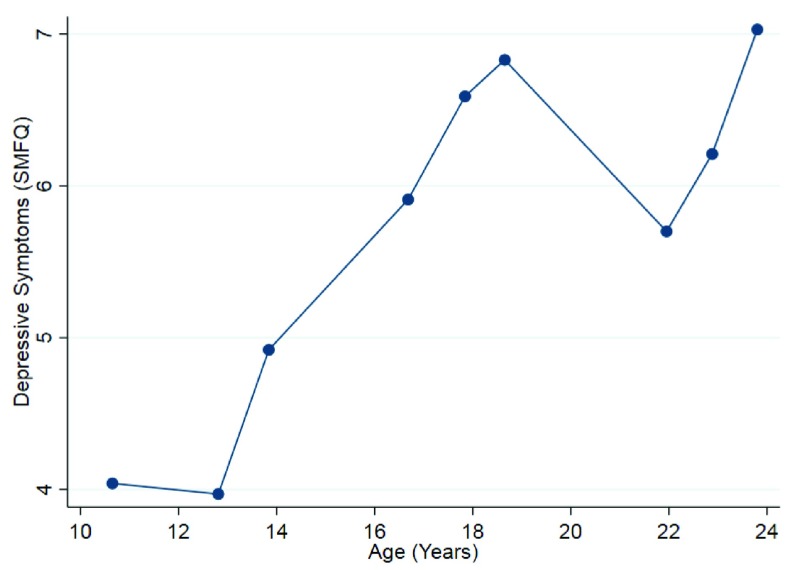
The overall pattern of depressive symptoms as measured by the Short Mood and Feelings Questionnaire (SMFQ) in ALSPAC.

One of the benefits of assessing the SMFQ repeatedly over time is the ability to examine the nature of depressive symptoms across multiple stages of development (i.e., late childhood to adolescence, across adolescence, adolescence to young adulthood). Table 3 and Figure 1 both highlight how the SMFQ has changed over time. From initially low levels of depressive symptoms in late childhood, scores tend to increase until the age of 18. From here, depressive symptoms begin to decline until the age of 22, where symptoms then begin to rise again to greater levels than previously observed at age 18. There is much more heterogeneity around the data towards the later stages of data collection with higher standard deviations observed. Likewise, the median and interquartile range tend to increase throughout the latter waves. Figure 2 shows histograms for the nine occasions of the SMFQ. The scores tend to be skewed towards smaller values across all occasions. However, there is a trend with the tails from the histograms getting larger across time as the distribution of scores slowly move towards the tails. Relatedly, the number of individuals scoring 11 or above on the SMFQ also tends to increase over time as shown in Table 3.

### Validity and utility of the SMFQ

Within ALSPAC, the SMFQ has good internal reliability as assessed by Chronbach’s alpha. Table 3 shows that the reliability is lowest on the first occasion (0.797, and highest on the seventh occasion (0.915). There are also strong correlations observed between each of the assessments ( *P* values < 0.0001). As Table 4 shows, there tends to be a pattern where occasions measured more closely together have higher correlations (i.e., ages 10.65 and 12.81, compared to the correlations between ages 22.88 and 23.8), and these are particularly strong towards the last three assessments ( *r* > 0.569). The strong correlation between all the assessments indicates that the SMFQ is a valid tool for examining depressive symptoms over time within ALSPAC.

**Table 4.** Table of correlations between all Short Mood and Feelings Questionnaire (SMFQ) results.

	SMFQ at age 10.65	SMFQ at age 12.81	SMFQ at age 13.84	SMFQ at age 16.68	SMFQ at age 17.84	SMFQ at age 18.65	SMFQ at age 21.95	SMFQ at age 22.88	SMFQ at age 23.8
**SMFQ at** **age 10.65**	-								
**SMFQ at** **age 12.81**	0.361 *	-							
**SMFQ at** **age 13.84**	0.271 *	0.528 *	-						
**SMFQ at** **age 16.68**	0.233 *	0.349 *	0.397 *	-					
**SMFQ at** **age 17.84**	0.202 *	0.297 *	0.365 *	0.502 *	-				
**SMFQ at** **age 18.65**	0.180 *	0.290 *	0.328 *	0.490 *	0.544 *	-			
**SMFQ at** **age 21.95**	0.178 *	0.268 *	0.283 *	0.406 *	0.424 *	0.454 *	-		
**SMFQ at** **age 22.88**	0.187 *	0.260 *	0.301 *	0.424 *	0.396 *	0.466 *	0.618 *	-	
**SMFQ at** **age 23.8**	0.171 *	0.264 *	0.320 *	0.409 *	0.402 *	0.462 *	0.569 *	0.664 *	-

* *P* <0.0001.

### Demographics of the SMFQ

A brief exploration of these data shows that the demographic information of individuals who have completed at least one assessment of the SMFQ varies from those who have not completed any assessments. Table 5 highlights these differences, but it is important to note that individuals without SMFQ measures are more likely to be male, have mothers with poorer educational attainment and lower socioeconomic status at birth, be the thirdborn or later child and have a younger mother.

**Table 5.** Participant demographics for individuals with at least one measurement of the Short Mood and Feelings Questionnaire (SMFQ).

Variable	Included in analysis, n (%)	Excluded from analysis, n (%)	*χ* ^2^	*P*
**Sex (n=14,854)**				
Males	4,495 (47.9)	3,140 (57.5)	128.98,	<0.001
Females	4,899 (52.1)	2,320 (42.5)		
**Maternal education (n=12,493)**				
A-level or higher	3,453 (40.9)	957 (23.7)	566.51	<0.001
O-level	2,380 (35.3)	1,347 (33.3)		
<O-level	2,016 (23.8)	1,740 (43.0)		
**Maternal socioeconomic status (n=10,118)**				
Professional/managerial/technical	2,940 (40.8)	841 (28.8)	126.95	<0.001
Skilled non-manual or lower	4,263 (59.2)	2,074 (71.2)		
**Parity (n=13,124)**				
First born	3,918 (45.9)	1,955 (42.5)	54.16	<0.001
Second born	3,041 (35.7)	1,547 (33.7)		
Third born or later	1,569 (18.4)	1,094 (23.8)		
**Maternal age at pregnancy (n=14,076)**				
<25 Years	1,531 (17.3)	1,830 (35.2)	660.82	<0.001
25–29	2,752 (31.0)	1,587 (30.5)		
30–34	3,201 (36.1)	1,272 (24.4)		
>35	1,388 (15.6)	515 (9.9)		

Pearson’s *χ* ^2^ tests used to highlight differences between participant demographics and individuals having at least one measure of the SMFQ.

## Considerations for the data

There are several considerations that should be noted when using the SMFQ data in ALSPAC. The first is that like all longitudinal studies, ALSPAC is subject to attrition and, as shown in Table 3, the sample size for using the SMFQ tends to decrease over time. As ALSPAC has a plethora for sociodemographic information and a number of other psychiatric assessments, it is possible to impute the missing data (for examine using multiple imputation with missing at random assumptions). Other longitudinal studies have used full information maximum likelihood to address patters of missing data, but considerations should be given to the issue of missing data when using the SMFQ.

The second consideration is that exploring the distribution of data revealed an anomaly in the data, with a random spike occurring at the fifth assessment of the SMFQ (age 17.84). A closer inspection of this data revealed that 183 individuals answered “sometimes” to every question of the SMFQ at this age. Sensitivity analyses in one recent study found that removing these individuals had no effect on the interpretation of the results ^2^. Still, researchers may choose to remove these individuals from analysis.

The final consideration is that future assessments of the SMFQ may become available within ALSPAC throughout the duration of the study. A tenth occasion will be released shortly which will address depressive symptoms around the age of 26. If ALSPAC continues to assess the SMFQ past this age, this study will be one of the few longitudinal studies with repeated assessments of depressive symptoms, along with a host of exposure and outcome data. It is also important to highlight that ALSPAC has other measures of depressive mood such as the DAWBA ^22^ (assessed at ages 7, 10, 13 and 15) and the CIS-R ^23^ (assessed at ages 18 and 24). Together, these data will be vital for exploring the nature of depression across multiple periods of development.

## Ethical approval and consent

Ethical approval for the study was obtained from the ALSPAC Ethics and Law Committee and the Local Research Ethics Committees, full details of the approvals obtained are available from the study website ( http://www.bristol.ac.uk/alspac/researchers/research-ethics/).

## Data availability

### Underlying data

ALSPAC data access is through a system of managed open access. The steps below highlight how to apply for access to ALSPAC data.

1. Please read the ALSPAC access policy which describes the process of accessing the data in detail, and outlines the costs associated with doing so.
2. You may also find it useful to browse the fully searchable research proposals database, which lists all research projects that have been approved since April 2011.
3. Please submit your research proposal for consideration by the ALSPAC Executive Committee. You will receive a response within 10 working days to advise you whether your proposal has been approved. If you have any questions about accessing data, please email alspac-data@bristol.ac.uk.

### Extended data

Open Science Framework: SMFQ-ALPSAC. https://doi.org/10.17605/OSF.IO/8TVGY ^21^.

This project contains the following extended data:

- ALSPAC Depression - Supplement.docx (Stata code used to create summary scores, Word file).
- create SMFQ.do (Stata code used to create summary scores, Stata file).
- Syntax.txt. (Stata code used to create summary scores, text file).

Extended data are available under the terms of the Creative Commons Zero “No rights reserved” data waiver (CC0 1.0 Public domain dedication).
