# Supplementary material for: Examining the longitudinal nature of depressive symptoms in the Avon Longitudinal Study of Parents and Children (ALSPAC)
Source: Wellcome Open Res. 2019 Oct 4;4:126. Originally published 2019 Aug 22. [Version 2] doi: 10.12688/wellcomeopenres.15395.2 (PMC6764237; doi:10.12688/wellcomeopenres.15395.2)
Supplement: Peer review history

# Peer review: Reviewer response for version 2

**DOI:** 10.21956/wellcomeopenres.16962.r36661

**Published:** 2019-10-07

**Authors:** Myrna Weissman

**Affiliations:** 1 Vagelos College of Physicians and Surgeons and New York State Psychiatric Institute, Columbia University, New York, NY, USA

Thank you for adding the material.

I confirm that I have read this submission and believe that I have an appropriate level of expertise to confirm that it is of an acceptable scientific standard.

# Peer review: Reviewer response for version 2

**DOI:** 10.21956/wellcomeopenres.16962.r36660

**Published:** 2019-10-04

**Authors:** Glyn Lewis

**Affiliations:** 1 UCL Division of Psychiatry, Faculty of Brain Sciences, University College London, London, UK

Thank you for the additional material.

I confirm that I have read this submission and believe that I have an appropriate level of expertise to confirm that it is of an acceptable scientific standard.

# Peer review: Reviewer response for version 2

**DOI:** 10.21956/wellcomeopenres.16962.r36554

**Published:** 2019-10-04

**Authors:** Stephan Collishaw

**Affiliations:** 1 Developmental Psychiatry Section, Division of Psychological Medicine and Clinical Neurosciences, School of Medicine, Cardiff University, Cardiff, UK

Depression is one of the most common mental health problems affecting young people. Depression is associated with major societal and individual burdens, including wide-ranging impacts on young people’s distress, peer and family relationships, education and long-term health. A developmental perspective is essential given the rise in the incidence of depression across adolescence and young adulthood, emerging gender differences, and a need to understand underlying risk and protective mechanisms in order to inform effective prevention.  

 This data note provides a helpful and informative guide to the use of the short Mood and Feelings Questionnaire (sMFQ) in the Avon Longitudinal Study of Parents and Children (ALSPAC). The sMFQ is a widely used and well-validated measure of depression symptoms. Repeated assessment of depression across nine occasions spanning late childhood, adolescence and early adulthood is a major strength of the ALSPAC cohort, and this facilitates longitudinal investigation into depression across this crucial developmental period. The use of the same measure on each occasion is a unique strength facilitating application of trajectory-based methods of analysis including across the transition to adult life. The data note provides important background to the measure, helpful descriptive data, and useful practical help for researchers planning to use the measure (e.g. inclusion of variable names, syntax for scoring, missing data patterns).

 A few suggestions for revision:

 There are well-established gender differences in depression, and these change across development. I strongly recommend providing a breakdown of sMFQ scores in ALSPAC by gender and age.

 Include reflection on the validity of self-reports (vs other informant reports) of depression, and whether this changes across age.

 Comment on whether differences in the mode of assessment (postal questionnaire vs research clinic) and variation in the gaps between assessments might affect analysis, and if appropriate provide recommendations.

 Indicate proportion of item-level missing data within measurement occasions

 Change heading “Demographics of the SMFQ” to “Predictors of response”

 Table 3: what is the age range of participants at each assessment?

 There are some typographical and grammatical errors (e.g. online para 2, line 8; para 4, line 1; para 6, line 10; para 8, line 2; Table 2, bottom row)

I confirm that I have read this submission and believe that I have an appropriate level of expertise to confirm that it is of an acceptable scientific standard.

# Peer review: Reviewer response for version 1

**DOI:** 10.21956/wellcomeopenres.16825.r36295

**Published:** 2019-09-26

**Authors:** Glyn Lewis

**Affiliations:** 1 UCL Division of Psychiatry, Faculty of Brain Sciences, University College London, London, UK

This is a useful and comprehensive account of the data on depressive symptoms available in the Avon Longitudinal Study of Parents and Children (ALSPAC) birth cohort study based in Bristol, UK. It provides a helpful background to investigators who want to use the data as well as providing some descriptive statistics on the cohort.

 It would also be helpful to have the median, interquartile range and percentage reaching a cutoff in Table 3 given the non-normal distribution of the SMFQ scores. It might also be useful to mention that there are other measures of depressive symptoms available in ALSPAC. These are the DAWBA assessments in childhood and adolescence and the CISR at 18 and 25 years.

I confirm that I have read this submission and believe that I have an appropriate level of expertise to confirm that it is of an acceptable scientific standard.

# Peer review: Reviewer response for version 1

**DOI:** 10.21956/wellcomeopenres.16825.r36354

**Published:** 2019-09-02

**Authors:** Myrna Weissman

**Affiliations:** 1 Vagelos College of Physicians and Surgeons and New York State Psychiatric Institute, Columbia University, New York, NY, USA

The Avon Longitudinal Study of Parents and Children [ALSPAC] is a unique longitudinal inter generational study of parents and children. Beginning with a cohort born between 1991/92 the sample has over 14000 children over 9 assessments with a 10 ^th^ ongoing. The assessments are not at regular intervals, likely depending on funding, but they are sufficiently regular to give life course information from childhood to young adulthood. Data are currently available up to age 24 years.The intergenerational aspect of this study makes it important as increasing data supports the strong relationship between parent and offspring psychiatric problems. Moreover, offspring of parents with major depression or other disorders are at high risk for one themselves. Thus, it is possible to see the early signs and symptoms and use this information for early interventions and prevention. Studies of resilience are also possible and biological studies such as MRI may be able to determine traits that run in families even if the offspring is not affected.

 In this paper data are presented for access to a 13 item assessment for depressive symptoms called Short Mood and Feelings Questionnaire SMFQ. The sample and access and use of this scale are very well described  for use by qualified investigators. The sample sizes, validity, and location of data etc. are included. The language and information are clear, and this will be of value to many investigators.

 The drop off in sample across the 9 waves is a problem in longitudinal studies and does seem to be a problem here. While the author describes ways of handling the fall off such as multiple imputation or maximum likelihood, it is essential to understand the demographic characteristics of those who remain and those who leave the sample. The author notes that males are less likely to have SMFQ scores.Thus data should be presented by gender. 

 This is a useful paper for investigators wanting to obtain this type of information and the presentation is well done.

I confirm that I have read this submission and believe that I have an appropriate level of expertise to confirm that it is of an acceptable scientific standard.

# Response

**Published:** 2019-09-04

**Authors:** Alex Kwong

I thank the reviewer for these comments and for the speed in reviewing this data note. I completely agree with the reviewer that missing data is a problem within ALSPAC and that understanding the demographics regarding participation (especially for depressive symptoms data) is important. That said, I am glad the reviewer shares my opinion that the depressive symptoms data in ALSPAC will be useful in studies to come and I hope this data note aids future researchers.
